# Toward a Long‐Term Low Emission Development Strategy: The Case of Energy Transition in Qatar

**DOI:** 10.1002/gch2.202200229

**Published:** 2023-05-01

**Authors:** Fadi Al‐Noaimi, Tareq Al‐Ansari, Yusuf Bicer

**Affiliations:** ^1^ Division of Sustainable Development College of Science and Technology Hamad Bin Khalifa University Qatar Foundation Doha Qatar

**Keywords:** decarbonization, emission development strategy, energy transition, low‐carbon development

## Abstract

The objective of this paper is to present a comprehensive perspective on the development of a long‐term low‐emission development strategy for Qatar, in line with the Paris Agreement. The methodology used in this paper takes a holistic approach by analyzing national strategies, structures, and mitigation measures from other countries, and synthesizing these with Qatar's unique context in terms of its economy, energy production, and consumption, as well as its energy‐related emission profile and characteristics. The findings of this paper identify key considerations and elements that policymakers would need to take into account when developing a long‐term low‐emission vision for Qatar, with a particular emphasis on its energy sector. The policy implications of this study are significant for policymakers in Qatar, as well as for other countries facing similar challenges in their transition to a sustainable future. This paper contributes to the discourse on energy transition in Qatar and provides insights that can inform the development of potential routes to reduce greenhouse gas emissions in Qatar's energy system. It serves as a foundation for further research and analysis and can contribute to the development of more effective and sustainable policies and strategies for low‐emission development in Qatar and beyond.

## Graphical abstract

This paper presents a holistic perspective to develop a long‐term low‐emission strategy for Qatar. It synthesizes several national strategies and considers Qatar's unique context, identifying key considerations for policymakers. The policy implications of this study are significant for Qatar's energy transition. The insights elicited from this research can inform more effective strategies for climate policy and low‐carbon development in Qatar and beyond.

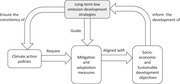

## 1 Introduction

Greenhouse gas (GHG) emissions have been on an upward trend since the industrial revolution, with significant increases in the past few decades.^[^ ^1^ ^]^ This increase in emissions has led to global warming and climate change, with adverse effects on the environment, human health, and economies. The economic and societal consequences are expected to rise the longer we wait to take strong action to cut emissions on a consistent basis.^[^ ^2^, ^3^ ^]^ The urgency of the situation has led to the adoption of the Paris Agreement in 2015, which aims to limit global warming to below 2 °C and pursue efforts to limit the temperature increase to 1.5 °C. The Paris Agreement also calls for countries to develop and communicate their nationally determined contributions (NDCs) toward reducing their GHG emissions. The NDCs reflect the countries' commitments to reducing emissions, which can be achieved through various measures such as decarbonizing the economy, increasing energy efficiency, and shifting to cleaner energy sources.^[^ ^4^ ^]^

**Figure 1:**
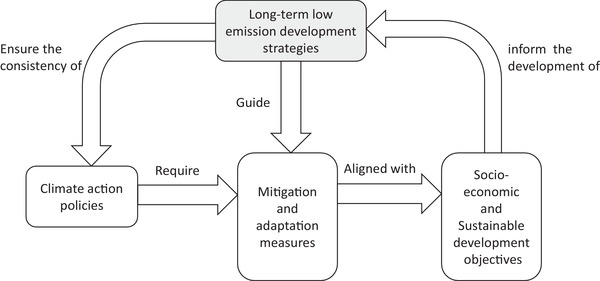
Interrelation between long‐term lowmission development strategies (LEDS), climate action, and sustainable development.

All signatories to the Paris Agreement are also expected, in line with Article 4.19, to design and publish their “long‐term greenhouse gas emission development strategies,” hereafter referred to as low‐emission development strategies (LEDS), that take into account commonalities and differences in responsibilities, capacities, and national contexts. LEDS typically needs to cover areas like mitigation, adaptation, and sustainable development that are intricately tied to the national socioeconomic system as a whole.^[^ ^5^ ^]^ 2008 saw the emergence of the concept of LEDS in the run‐up to the climate negotiations in Copenhagen (COP15) ushering an era in which the importance of these strategies is recognized as critical to both sustainable development and GHG emissions reduction, as embraced by the Copenhagen Accord.^[^ ^6^ ^]^ They act as a bridge between the national short‐term NDCs and the long‐term goals of the Paris Agreement, thus creating the framework for long‐term planning for both climate‐resilient and prosperous economies.^[^ ^7^ ^]^ As opposed to short‐term NDCs that need to be successively updated, LEDS set long‐term objectives for climate, social, and economic transformation while also guiding short‐term decisions to mitigate climate change. Long‐term strategies can provide a stable policy framework that enables countries to achieve emission reduction targets efficiently and cost‐effectively, while also addressing other development priorities such as poverty reduction, energy security, and economic growth.^[^ ^8^ ^]^ The significance of LEDS arises from its contribution to the complex climate change discourse at a national level, and its role in guiding the structural changes and transformation necessary in the socio‐economic systems to achieve the required decarbonization and emission reduction targets. As illustrated in **Figure** **1**, as a starting point, the wider goals of socioeconomic development, together with the emission reduction targets, should inform the formulation of these strategies to effectively align them with the national policy process and understand the impact of different climate policy options on broader national development. Additionally, LEDS provide the necessary cohesion in the administration of national development goals against the backdrop of trade‐offs and synergies, making them a vital component of every country's climate strategy.

Global climate change will have an impact on Qatar's environment, economy, and possibly even its politics and security. Qatar is especially vulnerable to the effects of global warming due to a lack of arable land and water resources, which are needed to develop sufficient carbon sinks, forests, and other green areas.^[^ ^9^, ^10^ ^]^ Its economic growth goals will be compromised if it is unable to strengthen its resilience to the effects of climate change. Qatar's stand on climate and any envisaged transformation plans are inextricably linked to its energy system. The narrative on the effects of energy transition in Qatar has been on the possible negative impact of decreasing revenues on economies. However, this discussion must be far broader. Since national development priorities and a long‐term vision for putting Qatar's energy industry on a more sustainable route are intertwined, it is imperative that Qatar formulate a long‐term emissions reduction strategy.

To that end, the purpose of this paper is to enrich the discourse on energy transition in Qatar and present a perspective that will help inform policymakers in Qatar while developing potential routes to reduce GHG emissions in Qatar's energy system and formulating its anticipated long‐term LEDS in accordance with the Paris Agreement. While various studies have explored methods to reduce emissions and decarbonize energy systems, to our knowledge, there has been a lack of research that specifically addresses the development of long‐term LEDS in the context of Qatar. Our paper takes a holistic approach by analyzing selected national strategies, structures, and mitigation measures from other countries, and synthesizing the gained insight with the specific context of Qatar's economy, energy production, and consumption, as well as its energy‐related emission profile and characteristics. Rather than developing a specific strategy, this paper aims at identifying key considerations and elements that policymakers would need to take into account when developing a long‐term low‐emission vision for Qatar with a particular emphasis on its energy sector. By tailoring our approach to the specific circumstances of Qatar, our aim is to facilitate a more structured discourse and assessment of the design, process, and implementation of LEDS. Through our paper, we intend to provide a comprehensive perspective on the factors that need to be considered for such strategies and how these can be tailored to the unique characteristics of Qatar's energy system. This, in turn, can serve as a foundation for further research and analysis, and contribute to the development of more effective and sustainable policies and strategies for low‐emission development in Qatar and beyond. This study has significant implications for policymakers in Qatar, as well as for other countries facing similar challenges in their transition to a sustainable future.

The remainder of this paper is organized as follows: Section 2 provides a brief overview of the latest studies in the field of low‐emission development measures and strategies, with a specific focus on research conducted in Qatar. This section aims to identify gaps in the literature and possible research directions that can contribute to the development of a long‐term low‐emission development strategy for Qatar. Section 3 sets the stage for the national emission strategies by reviewing economy‐wide LEDS adopted by different nations with emphasis on energy system mitigation measures and low‐carbon technologies in particular. The insights elicited regarding how these countries aim to restructure their energy systems toward low energy‐related emissions (and, in most cases, net‐zero emissions) are evaluated and discussed. Section 4 focuses on the context of Qatar by first studying its energy sector and energy‐related emissions characteristics. In Section 5, policy implications for the design, process, and implementation of LEDS in Qatar are presented. Finally, Section 6 concludes the paper with the limitations of this research and suggested future research directions.

## 2 Literature Review

The literature review in this paper begins with a global perspective on emission reduction strategies and policies by providing a broad overview of the current state of research in this field. The scope is subsequently narrowed down to the context of Qatar, with a focus on examining relevant research related to energy‐related emissions reduction, decarbonization strategies, as well as energy transition and low‐carbon development at large.

Limited research has been undertaken to investigate long‐term emission reduction strategies at a national level, with a focus on energy‐related emissions. A broad effort established a framework for the design of sixteen national mid‐century low‐emission pathways compatible with the Paris Agreement as part of the Deep Decarbonization Project.^[^ ^11^ ^]^ Focusing on the trends, policies, and pathways of long‐term low‐carbon development, an in‐depth analysis of China's actual national circumstances and development characteristics was carried out and investigated emission reduction pathways, technology support, and costs against the backdrop of the long‐term goal of deep decarbonization.^[^ ^12^ ^]^ Another study developed six long‐term scenarios for China and performed a quantitative evaluation of each scenario's carbon emission pathways, energy transformation, technology, policy, and investment requirements while taking into account the carbon‐neutrality objective of China as well as temperature‐related constraints specified by the Paris Agreement.^[^ ^13^ ^]^ Similarly, a comparative analysis of twenty‐six distinct scenarios aimed at transforming energy systems in Germany was conducted, with the ultimate objective of reducing CO_2_ emissions by at least 90% by the year 2050; the study highlighted the lack of consensus on what constitutes the most effective strategy.^[^ ^14^ ^]^

At a broader level, other literature has highlighted the significance of *inter‐relationships* between GHG emissions, energy intensity, and economic growth at the national level. A recent study investigated the cause‐and‐effect relation between CO_2_ and indicators like energy use, gross domestic product (GDP), and employment between 1995 and 2019 for a group of eight countries in south‐east Europe in the context of shifting to low‐carbon development to provide more policy insight into these countries' sustainability pathways.^[^ ^15^ ^]^ Another effort examined the complex and ever‐changing links between energy consumption, land concentration, CO_2_ emissions, and economic growth in China at various stages of development of its economy.^[^ ^16^ ^]^ Similarly, a study investigated the potential macroeconomic effects of reducing GHG emissions in Thailand from 2010 to 2050.^[^ ^17^ ^]^ The study used a computable general equilibrium (CGE) model to assess different GHG mitigation scenarios and reduction targets while employing econometric methodologies to examine the connections among variables that contribute to nationwide emissions. Another study constructed a theoretical framework to elucidate the association between the overall domestic expenditure and carbon dioxide emissions in Pakistan.^[^ ^18^ ^]^ Through this framework, a threshold was identified that played a significant role in shaping the country's carbon emissions from 1973 to 2018. In the context of the Green Deal, the dual relationship between economic growth and climate change measures was investigated.^[^ ^19^ ^]^ GHG emissions were estimated for three potential future states, including the current scenario with existing measures, an additional measures scenario, and a business as usual scenario using a novel predictive approach. The results showed that the Baltic States' climate policies were not sufficient to meet the emission reduction targets set for 2030.

With a focus on Qatar's energy sector transition, **Table** **1** provides a summary of energy‐related GHG emissions reduction measures and decarbonization. It is evident that there is a significant lack of comprehensive and forward‐looking research pertaining to the development of a long‐term low‐emission strategy for Qatar. While virtually all decarbonization topics and cross‐cutting measures have been covered, the majority of the existing literature focuses on individual elements of Qatar's energy system and is limited to investigating the potential of technology deployment or alternative energy carriers, as well as decarbonization options. Far from being holistic, the literature's focus has been confined to either specific sub‐sectors or technology within Qatar's broader energy system. Examples include the electrification of transport, the potential of renewables, and hydrogen production and export. Conversely, the use of energy in industry and its related emissions have received little attention from scholars and therefore our research is particularly pertinent and relevant. Our study fills an important gap in the current literature by providing a comprehensive and forward‐looking analysis specific to the Qatari context and its energy sector in particular. To that end, we highlight later in Sections 5 and 6 several gaps that are suggested to be addressed in future research directions in the context of energy transition and long‐term low‐emission development initiatives as particular to Qatar's energy and economy.

**Table 1:** Summary of literature review in the context of energy in Qatar

Focus	Study	Objective
Renewables	[20]	Analyzed the potential of integrating renewable energy into Qatar's power grid.
	[21]	Quantified the potential for solar energy penetration by modeling Qatar's electrical grid expansion over the following decades.
	[22]	Investigated the potential of wind energy in Qatar and assessed GHG emissions reduction from displaced fossil‐based electricity of a case study of three wind farms.
Electrification	[23]	Analyzed the viability of using electric vehicles in Qatar from an economic and practical standpoint and underlined the need for incentives and significant subsidies for uptake.
	[24]	Developed a life cycle assessment to quantify the environmental impacts of electric mobility in Qatar including social and economic indicators.
	[25]	Studied the optimal configuration of 50 renewable‐based electric vehicle fast charging stations to satisfy daily demand.
Hydrogen	[26]	Assessed Qatar's capacity and its energy sector's competitiveness in producing and exporting hydrogen.
	[27]	Investigated the potential of decarbonization in industry using a green hydrogen via optimally sizing hydrogen production across hydrogen supply chain.
	[28]	Analyzed a number of potential hydrogen production alternatives in Qatar.
Carbon capture, use and storage	[29]	Proposed a carbon capture and use (CCU) network integrated into industrial processes and assessed its economic viability.
	[30]	Examined the critical carbon credit pricing to determine at what price carbon capture and storage (CCS) system investments are most profitable.
	[31]	Developed mixed integer programming model to integrate CCS to differnt sinks under economic and environmental constraints in allocation/scheduling problem.
Sector‐wide	[32]	Developed an optimization model covering a range of sectors covering Qatar's energy infrastructure for planning and policymaking insight.

## 3 Research Methodology

By situating our study within the broader global context and then delving into the specific context of Qatar, we provide a comprehensive and nuanced analysis of emissions reduction strategies that take into account the unique circumstances of the country. This approach allows us to identify key considerations and elements that policymakers in Qatar must take into account in developing a long‐term low‐emission vision for the country, while also contributing to the broader literature on emission reduction strategies and policies. Fittingly, we expanded our analysis to include national long‐term low‐emission strategies for several countries as will be further elaborated in the following section.

We analyze and compare national long‐term low‐emission development strategies for selected nations, with a particular emphasis on the underlying structures and aspects that support mitigating actions and measures to reduce energy sector‐related GHG emissions. Our aim is to identify common themes and characteristics that could inform Qatar's own low emission development strategy for its energy sector. This methodological approach is founded on the assumption that there are valuable insights to be gained from the experiences of other nations that have already embarked on comparable, and even more aggressive, initiatives to reduce emissions and transition toward a low‐carbon economy. To achieve this objective, the study first identifies a sample of countries that have already established comprehensive long‐term low‐emission development strategies. The selection of the sample is guided by a set of criteria that reflect the relevance and similarity of the countries' economic, political, and social contexts to that of Qatar. The countries' strategies are then analyzed using a framework that enables the identification of recurring themes and characteristics across different aspects of the low‐emission development strategies. To better understand the structure, essential elements, commonalities and differences, scope, and coverage of LEDS, this section critically examines long‐term strategies for selected nations that have already developed their LEDS and submitted them to the United Nations Framework Convention on Climate Change (UNFCCC). Our analysis aims to highlight and discuss the most salient themes that emerge from our review. This is motivated by the necessity that Qatar's strategy to low‐carbon development must be planned and shaped in light of the global strategic context of GHG emissions reduction efforts. Hence, this analysis is presented to assist policymakers in Qatar while formulating Qatar's national mid‐century low‐emission development strategy. This is, however, by no means an exhaustive assessment of all submitted strategies; rather, it is an analytic commentary on a representative set of strategies with the goal of highlighting important themes and eliciting relevant insights and lessons from them.

The selection of national strategies for review aims to demonstrate a greater diversity of national perspectives. As of September 2022, 51 parties to the Paris Agreement officially submitted their LEDS to UNFCCC.^[^ ^33^ ^]^ Ten countries that formally submitted their LEDS were selected representing about 49% of global GHG emissions as shown in **Table** **2**. Five of the top ten emitting nations' strategies were selected (China, USA, Indonesia, Japan, and Canada). As for the other five highest‐emitting countries (India, Russia, Brazil, Iran, and Saudi Arabia), no official submission of their long‐term emission development policies was noted at the time of conducting the current review. We included both developing and developed economies, with South Africa standing in for the former and Germany and the UK for the latter. Norway and Australia were included in the selection in order to take advantage of any conceivable parallels between Qatar's energy profile, being a major natural gas exporter, and those of other exporting nations whose strategies were developed. Additionally, the nations' vulnerability to climate change was also factored into our selection to cover a wide range extending from very vulnerable countries such as Japan and Germany to much less vulnerable countries as in the case of Norway. The Germanwatch institute's Global Climate Risk Index (CRI) 2020, presented at COP25 in Madrid in 2019, was used to assess nations' exposure and vulnerability to extreme weather occurrences. The lower the score, the greater a country's vulnerability to climate‐related severe events.^[^ ^34^ ^]^

**Table 2:** Selected countries' contribution to the global GHG emissions and vulnerability to climate change^[^ ^34^, ^35^ ^]^

Country	Share of global emissions	CRI score
China	24%	45.17
United States	12%	23.83
Indonesia	3.5%	76.83
Japan	2.4%	5.50
Canada	1.6%	21.83
Germany	1.6%	13.83
Australia	1.3%	49.50
South Africa	1.1%	53.33
United Kingdom	0.9%	73.83
Norway	0.06%	138.83
Total	48.46%	–

The Paris Agreement has not set a standard format or structure for LEDS, nor has it outlined a breakdown of the aspects that must be addressed throughout their development. To accomplish its intended function as a way for nations to explore emission reduction pathways or even carbon neutrality, LEDS would be required to have a number of critical components.^[^ ^36^ ^]^ In the subsequent discourse, we discuss three fundamental elements that hold significance in developing LEDS: economy‐wide vision and sector‐specific targets; scenario‐based mitigation; and energy sector in LEDS. Corresponding details are illustrated in **Table** **3**.

**Table 3:** Long‐term low emission development strategies

Emission target	Scope	Governance	Mitigation models	Mitigation measures and targets			
				Power	Industry	Transport	Buildings
China^[^ ^37^ ^]^ Carbon neutrality before 2060	All GHGs	Policy	✗	Share of non‐fossil energy: • (2030) 25% • (2060) 80% • (2030) 1200 GW Advanced nuclear energy	Electrification CCUS in steel, cement, and chemicals	(2030) 40% of new vehicles: (PEV, hydrogen, NG) Reductions (from 2020 level): • 9.5% carbon intensity • 10% energy consumption per unit Advanced biofuels	(2025) 100% of new buildings green standards compliant (2025) 50% of public buildings covered with rooftop PV (2025) 8% of fossil fuel replaced by renewables
USA^[^ ^38^ ^]^ (2030) 50–52% below 2005 levels (2050) Net‐zero emissions	All GHGs	Policy	✓	(2035) 100% clean electricity	Electrification of Low and medium heat Incentives : • Clean hydrogen • Waste to power • CCS	(2030) 50% of sold light vehicle: zero emission (2030) 3 billion gal of SAF EV charging infrastructure Scaling up biorefineries	Electrification Energy efficiency Building energy codes
Indonesia^[^ ^39^ ^]^ (2060 or sooner) net‐zero emission	All GHGs	No political pledge	✓	(2050) 43% renewables in power mix (2050) 100% renewables in remote areas (2050) 8% BECCS in power mix (2050) 271 GW of installed renewables (2050) 76% of biomass‐coal cofiring plants with CCS (2050) Carbon intensity at 104 g CO per kWh	CCUS CO_2_ cap Natural gas replacing coal Hydro Power (in smelters) Electrification Energy efficiency	(2050) Biofuels share: 46% (2050) Electrification share: 30% Energy efficiency	Electrification Energy efficiency
Japan^[^ ^40^ ^]^ (2030) 50% of 2013 levels (2050) net‐zero emissions	All GHGs	Law	✗	(2030) 59% share of non‐fossil in power mix Nuclear power Energy storage systems Hydrogen and ammonia as fuel CCUS	Hydrogen in steel CCS in cement High heat process: • Hydrogen • Synthetic methane • Biomass	Electric vehicles Fuel cell vehicles Synthetic fuels	Energy efficiency Renewable heat Electrification
Canada^[^ ^41^ ^]^ (2050) net‐zero emissions (2030) 40– 45% below 2005 levels	All GHGs	Law	✓	Energy mix and renewables: • Hydro power • Nuclear energy • Wind and solar • Energy storage Retrofitting CCS	CCS Electrification Cogeneration Waste heat recovery Energy efficiency	Electrification Renewable natural gas District heating Energy efficiency Retrofitting building stock	

Emission target	Scope	Governance	Mitigation models	Envisaged sectoral mitigation measures and targets			
				Power	Industry	Transport	Buildings
Germany^[^ ^42^ ^]^ After (2050): Net negative emissions (2045) net‐zero emissions (2030) 65% of 1990 levels (2040) 88% of 1990 levels	All GHGs	Law	✗	(2030) Emission budget: 108 Mt CO_2_eq Renewables share in power mix: (2030) 80% (2035) 100% (2022) Shut nuclear plants (2038) Exit coal fired plants	(2030) Emission budget: 118 Mt CO_2_eq CCS Waste heat Circular economy	(2030) Emission budget: 85 Mt CO_2_eq 28% of demand is met from renewables Incentives for: • Electric cars • Green hydrogen • Biofuels from waste/residues Charing/fueling infrastructure for alternative fuels	(2030) Emission budget: 67 Mt CO_2_eq (2050) 80% reduction in demand (2005 levels) Electrification of heat supply (2050) climate‐neutral Buildings
Australia^[^ ^43^ ^]^ (2050) net‐zero emissions	All GHGs	Policy	✓	(2050) 90% below 2005 emissions levels Share of renewables: • (2050) 100% Clean electricity Energy storage Digital grids	(2050) 20% below 2005 emissions levels CCS and hydrogen 50% emission reduction from heavy industries Low emission technologies: Steel, aluminum, and cement	(2050) 50% below 2005 emissions levels Electrification Enabling infrastructure Alternative fuels	Energy efficiency
Electrification On‐site renewables							
South Africa^[^ ^44^ ^]^ (2050) 212–428 Mt CO_2_eq	All GHGs	Policy	✗	(2030) 38% share of renewables in power mix	(2030) 16% reduction in: energy consumption	(2030) 20% reduction in: road fleet energy intensity (2030) 20% reduction in: municipal fuel intensity Biofuels	(2030) Reduction in energy intensity: • Public buildings 50% • Municipal 20% Consumption: • New residential 33% • Existing residential 20% • Commercial 37%
United Kingdom^[^ ^45^ ^]^ (2050) net‐zero emissions	All GHGs	Law	✗	(2035) Clean electricity (2030) 40 GW offshore wind (2030) 1 GW offshore wind (2030) 5 GW hydrogen Small modular reactors Natural gas with CCS	(2030) 4 CCUS clusters Capturing 20‐30 Mt CO_2_ Inclduing 6 Mt CO_2_ of industrial emissions Carbon pricing Growing hydrogen, CCUS and renewables	(2030) End the sale of new petrol and diesel cars (2035) All new cars sales zero emissions capable (2040) Phase out diesel trains Electrification of rails/transit EV charging infrastructure (2030) 10% SAF delivery	(2028) 600 000 heat pumps (2035) New heating appliances are low‐carbon technologies (2035) No new gas boiler sold (2037) 75% emission reduction in public sector buildings Energy efficiency
Norway^[^ ^46^ ^]^ (2030) 50–55% below 1990 levels (2050) 80–95% below 1990 levels	Excludes F‐gases	Law	✗	Bioenergy with CCS Wind power	Electrification CCS Bioenergy Hydrogen as: • Reducing agent • Energy carrier Innovation and circularity	EU‐Effort Sharing Regulation (2021‐2030) annual emissions targets	EU‐Effort Sharing Regulation (2021‐2030) annual emissions targets

### 3.1 Economy‐Wide Vision and Sector‐Specific Targets

The premise of long‐term strategies is to establish a 30‐year planning horizon that is long enough to both guide the near‐term actions and enable an economy‐wide transformation to achieve decarbonization and compliant emission trajectories that are interlinked with sustainable development objectives, thereby integrating the climate change agenda into sustainable economic development in accordance with the Paris Agreement. While long‐term vision is primarily mitigation driven, it may also encompass mechanisms for increasing the nation's adaptation to climate change and resilience as well as reducing its vulnerability. The phrase “net zero” has taken a center stage of national discourse when considering the significant economic shift required to tackle climate change and, in particular, the necessary transformation in energy systems. While it is, in principle, up to countries to define their climate action depending on their national priorities, a rising number of countries have committed to achieving net‐zero emissions by the year 2050 as part of their efforts to reduce GHG emissions. Emission targets, whether economy‐wide or sector‐specific, are either expressly established in advance as visionary and referenced to a base year or are the result of modeling or scenario development.

Long‐term strategies typically focus on the sectors that are accountable for the bulk of emissions as determined by the nation's emission profile, such as energy, land use, industrial processes, agriculture, and waste. The emission profile of a country is used to identify which sectors should receive the most attention in a country's long‐term strategy. Profound changes envisioned in targeted sectors are facilitated by the set of policies and strategies aimed at reducing emissions across these sectors. This includes mitigation measures, technologies, and investments. To aid in achieving the economy‐wide goal of reducing emissions, LEDS may also incorporate sector‐specific mitigation targets with measurable metrics. These targets can be explicit emission reduction or non emission‐related targets as in the case of achieving higher efficiency in the energy sector or a higher share of renewables in the electricity generation mix.

As shown in Table 3, when presenting their targets to achieve net‐zero emissions by mid‐century, different terms were used. China used carbon neutrality to present its vision of becoming carbon neutral before 2060. The scope of China's strategy^[^ ^37^ ^]^ covered all types of non‐CO_2_ emissions. China's submitted LEDS established the strategy's guiding principles by linking both the schedule of carbon dioxide emission peaking before it starts to decline, and emission reduction to the country's economic and social development. It also conveys the emission intensity's binding target from the national policy plan, which is set at a 6% reduction from 2005 levels. The United States, on the other hand, has set a goal of “net‐zero emissions” by 2050. Along with the 2050 goal, the strategy includes short‐term targets from the updated 2030 NDCs and the US climate strategy.^[^ ^38^ ^]^ Its strategy asserts that the United States must take bold action over the next decade and relevant milestones must be met in the not‐too‐distant future to put the nation on a path to achieve economy‐wide net‐zero emissions by 2050. To that end, the US has pledged to reduce its total GHG emissions by 52–54% below 2005 levels by 2030 and 100% clean electricity by 2035.

Norway has not pledged to net‐zero emissions yet. It does, however, envision a low‐emission society.^[^ ^46^ ^]^ Norway plans to cut its GHG emissions by 50–55% by 2030 compared to what they were in 1990, and by 80–95% by 2050. The Climate Change Act of Norway specifies Norway's climate goals for 2030 and 2050. It serves as the foundation for its long‐term strategy, providing strong backing for its implementation. As for South Africa,^[^ ^44^ ^]^ emissions will begin to fall in absolute terms in 2036 to a range between 212 Mt CO_2_eq and 428 Mt CO_2_eq. Therefore, there is no commitment toward achieving net‐zero emissions by 2050 along with its strategy. South Africa's LEDS is based on government policies already in place to address mitigation across key sectors of the economy including energy. In 2019, the Climate Change Act of 2008 was passed in the United Kingdom, making it the first major economy to require net‐zero GHG emissions by the year 2050. UK's LEDS^[^ ^45^ ^]^ built on the government's 10‐point plan for a green industrial revolution kick‐started in 2020. In keeping with the Sixth Carbon Budget,^[^ ^47^ ^]^ the strategy established an indicative delivery pathway with projected emissions reduction across sectors for the period 2033 through 2037 and ultimately on the path to net zero by 2050.

### 3.2 Scenario‐Based Mitigation

Several of the studied LEDS contained modeling and mitigation scenarios in its structure as illustrated in Table 3. Economic models of various types and scopes are used to develop mitigation scenarios and pathways toward achieving the decarbonization and emission reduction targets communicated in the economy‐wide vision. These models are constructed with almost exogenous inputs, including assumptions about the prices of technology, the preferences of consumers, and the behaviors of the economy as well as emission drivers.^[^ ^48^ ^]^ While they can be informative, they are limited in the extent to which they can forecast the future. Instead of predicting the future, they provide an overview of a selection of policy and technological alternatives that may be combined to achieve the targeted emission trajectories. Nonetheless, strategies that are solely based on the output of modeling run the risk of being flawed and should be avoided, instead, a balanced approach should be adopted.^[^ ^49^ ^]^ China's strategy does not include any modeled scenarios and pathways for emission trajectories. Conversely, the US's LEDS makes extensive use of a wide variety of analytical tools, including a globally integrated assessment model that takes into account all GHGand economic sectors.^[^ ^38^ ^]^ This is supplemented with a variety of sensitivity scenarios for the emission trajectories toward the net‐zero target. Twelve scenarios depicting different pathways to net‐zero emissions based on underlying assumptions for the cost of technology, GDP growth, population, and energy prices were modeled. Similarly, modeling and analysis have been used to inform Australia's strategy to identify the technologies that Australia should focus on most, as well as how soon and to what extent the costs of these technologies may decline.^[^ ^43^ ^]^ McKinsey complemented this with top‐down economic modeling and bottom‐up analysis to assess the potential economic impacts. The outcome of the analysis shows a few potential technology options that might put Australia on track to achieve net‐zero emissions by 2050. No mitigation scenarios or pathways were included in South Africa's LEDS.^[^ ^44^ ^]^ It, however, indicates that the national GHG emissions pathway, as expressed in the national climate policy is used as the gauge to evaluate the strategy due to the lack of an agreed quantitative articulation of the vision. Canada's strategy outlined six modeled scenarios with varying emissionreduction aspirations suggesting that Canada's future clean electricity generation portfolio could take various forms with a considerable disparity in the projected demand growth and share of energy sources depending on the modeled scenario. To meet end‐use electrification requirements, Canadian electricity generation will expand significantly in all scenarios. Norway's LEDS did not include modeling of mitigation scenarios for emission reduction pathways.

### 3.3 Energy Sector in LEDS

**Figure 2:**
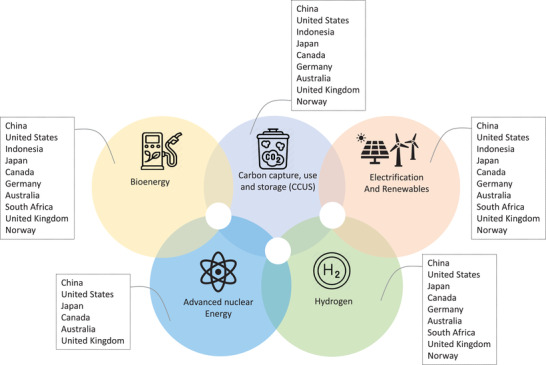
Decarbonization options in countries' LEDS.

The energy sector has a significant role in countries' efforts to achieve the goals of their LEDS since it is responsible for more than two‐thirds of global GHG emissions. Therefore, more emphasis is placed in this review on the mitigation efforts in the energy and industrial processes sectors and emission reduction targets aimed at energy‐related emissions at large. In the context of the energy transition, decarbonization options and mitigation measures enabled by the accelerated deployment of low‐carbon existing and emerging technologies, as well as innovation, have underpinned the majority of national initiatives aimed at achieving a low‐carbon transition. Still, all viable technologies and emissions reduction options are embraced by countries to varying degrees depending on associated costs, technology maturity, policy preferences, the market, and national circumstances. The key pillars of global energy system decarbonization identified by the International Energy Agency are energy efficiency, behavioral changes, and four technical value chains: renewables and electrification, carbon capture use and storage (CCUS), hydrogen and hydrogen‐based fuels, and bioenergy.^[^ ^50^ ^]^ The long‐term strategies looked at in this study mirrored countries' interest in examining different routes and capitalizing on the potential role of advanced clean technologies frequently identified in global energy transition scenarios and the reduction of energy‐related GHG emissions, as shown in **Figure** **2**. Insights regarding how these countries aim to restructure their energy systems toward low energy‐related emissions (and, in most cases, net‐zero emissions) are discussed in this section, and a summary of how countries intend to restructure their energy sectors and what mitigation measures are envisaged are illustrated in Table 3.

China's target of achieving carbon neutrality by 2060, as articulated in its NDCs to which the strategy is aligned, puts a primary focus on the energy sector, low‐carbon transformation in the industry sector, and acceleration of zero‐carbon industrial parks. Targets for the share of non‐fossil energy in the power mix are set at 25% in 2030 and 80% in 2060, respectively. By 2030, the installed capacity of wind and solar power will have expanded to over 1200 Gigawatt. As for the building sector, green and low‐carbon construction in urban and rural areas is encouraged. Green building standards will be implemented in 100% of new buildings in cities and towns by 2025. The rate at which renewable energies replace fossil fuels in urban buildings in China is targeted to reach 8% as the country speeds up its optimization of the building sector's energy use. Similarly, China plans to increase the use of electricity, hydrogen, natural gas, and advanced liquid biofuels in the transport sector. Other sector‐specific metrics are targeted in the transport sector for emission intensity and energy consumption, as illustrated in Table 2. In addition to energy efficiency, energy savings, and the circularity between energy production and consumption, China's LEDS lays out a variety of technical paths; end‐use electrification is seen as the primary path, made possible by infrastructure and the replacement of fossil fuel consumption. Another major focus is restructuring the power system to allow for greater use of advanced nuclear energy and renewable sources, as well as the use of alternative fuels like hydrogen and natural gas in hard‐to‐abate sectors. This will be supplemented by the rapid deployment of cost‐effective CCUS in the power generation, cement, steel, and chemical industries, as well as the recognition of the role of carbon removal. In addition to sector‐specific technology milestones, China's LEDS underlined the need for innovation and policy‐oriented measures in order to solve the complex decarbonization problem. Five key transformations were identified in the United States's LEDS for all routes of the energy transition to net‐zero: decarbonization of electricity, electrification, fuel switch, reducing energy waste, reducing methane and other non‐CO_2_ emiissions, and scaling up CO_2_ removal.

Four pillars were used to develop Indonesia's LEDS: Energy efficiency, clean electricity in buildings and transport, transition away from coal to renewables and natural gas, and penetration of renewables in energy end‐use sectors. The premises upon which Japan's LEDS is built are: increasing electrification in energy end‐use sectors through decarbonized electricity; energy efficiency; hydrogen‐reduced iron and steel making in the industry; use of renewable heat, hydrogen, synthetic methane, biomass where electrification is not possible; expansion of electric vehicles and fuel cell vehicles; and finally, where CO_2_ is unavoidable, the use of technologies such as bioenergy. The strategy projects that 59% of electricity will come from sources other than fossil fuels by 2050. Moreover,it suggests exploring the use of hydrogen and ammonia in power generation, which would require a more innovative, reliable, and cost‐effective supply chain. The strategy lacked both intermediate and long‐term sector‐specific emission reduction targets as well as mitigation models for potential emission pathways and scenarios. Not only does Canada's LEDS inform the overarching framework for Clean Growth and Climate Change in Canada, but it also illustrates potential emission reduction solutions, possible disruptive technologies, and areas where abatement will be more difficult and will necessitate a combination of policy and technology emphasis. It also examined alternative approaches Canada can take to achieve a low‐carbon economy by 2050 and identifies associated opportunities and obstacles. The guiding premise for decarbonizing Germany's electricity system is that all sectors must first significantly and permanently reduce their energy consumption (energy efficiency first). Second, insofar as it is feasible and economically viable, all end‐use sectors must use renewable energy directly. And third, if electricity from renewable sources is used for industry heat provision, transport, and other sectors, it must be used efficiently considering the increasing electrification and therefore electricity demand (sector coupling). Germany's long‐term strategy is underpinned by sectoral targets until the year 2030, identifying transformation pathways that are technology‐agnostic for all areas of action by actors and providing necessary guidance for investments with certainty.

Four factors are identified as crucial to the success of Australia's LEDS for the whole economy, in which technology will play a major role in achieving Australia's targets:^[^ ^43^ ^]^ lowering the costs of low‐emission technologies; the government's instrumental role in facilitating the deployment of new technologies by working closely with private industry; leveraging the global trend toward lower emissions while still meeting the needs of established markets; and collaborating with other nations to accelerate innovation in decarbonization technologies. The technologies targeted in the investment road map, incorporated in the strategy, may accomplish around half of the necessary reductions in emissions for reaching net‐zero emissions. Priority technologies include, ultra‐low‐cost solar, clean hydrogen, energy storage, low‐emissions steel and aluminum, and carbon capture and storage. The main mitigation measures envisioned by South Africa's strategy include decarbonization of the energy supply, diversification of the electricity generation mix, second and third generation biofuels, energy efficiency, solar water heaters, building regulations, and promotion of cleaner mobility. Cross‐cutting mitigation measures, such as carbon price, sectoral emission targets, carbon budgets, and the phasing out of fossil fuel incentives, were also emphasized in the long‐term strategy. The imminent need for planning and sector analysis to identify pathways and options is highlighted due to the absence of mitigation scenarios. A three‐stage process for the LEDS implementation is also detailed into short, medium, and long‐term phases. In the short‐term interval, a true transition is envisioned to start and be completed before the end of 2021. This is the case in areas where decarbonization is a viable alternative. The strategy may be put into action quickly to drive immediate reduction. During the medium‐term interval, new decision and investment criteria are generally used, resulting in changes to day‐to‐day operations to several sectors. In the last stage, low‐emission and decarbonization alternatives become the norm. Coordination of policy actions will be necessary for the successful implementation of all three phases of LEDS.

UK's LEDS posits that low‐cost, clean electricity would serve as the backbone of a future “net zero economy.” Renewables and state‐of‐the‐art small modular reactors are slated to become the backbone of Britain's electricity grid. By 2035, it is envisaged that the power system will be completely carbon‐free. While electricity will be the primary energy source, other low‐carbon fuels like hydrogen and biofuels are seen as important for the net‐zero strategy's success. New hydrogen and industrial carbon capture business models will receive government funding through revenue support schemes as part of an effort to scale up the production of cleaner fuels. The strategy outlined essential measures for the transformation required in the industry, including fair carbon pricing, expanding new industries in low carbon hydrogen alongside CCUS and renewables, and speeding up decarbonization in industrial clusters responsible for over half of the UK's emissions. As for heat and buildings, 2035 has been set as the deadline for replacing all existing residential and commercial heating systems with carbon‐neutral technologies, such as electric heat pumps and hydrogen boilers. A decision on the role of hydrogen in the heating system will be taken in 2026. The strategy envisions the zero emissions vehicle mandate as the catalyst for the transportation sector's transformation. Other transportation‐related decarbonization efforts include, among other things, infrastructure support for electric vehicles, rail electrification, and support for the commercialization of sustainable aviation fuels (SAF), and the development of SAF plants. Norway's strategy draws on an already‐established Norwegian industries roadmap that identifies the technologies and measures required for both emission reduction and growth including, carbon capture and utilization, bioenergy, hydrogen, innovation in production methods, circularity, and waste recovery. On its path toward a low‐emission society, Norway intends to achieve its enhanced emission targets by cooperating with the European Union on climate action based on three pieces of legislation; EU Emissions Trading System (EU ETS), Effort Sharing Regulation, and Land Use, Land‐Use Change and Forestry (LULUCF) Regulation. Industry and energy supply related emissions are governed by the EU ETSrepresenting more than 50% of Norway's total emissions. Transport and building emissions are mainly covered by the Effort Sharing Regulation. As for LULUCF regulation, it applies to, inter alia, land emissions accounting (beyond the scope of this study being non‐energy‐related emissions).

## 4 Discussion: The Context of Qatar

Qatar's 2021 NDCs were submitted to UNFCCC in November 2021, showing that the country is committed to reducing its GHG emissions. Qatar wants to cut its GHG emissions by 25% compared to a baseline scenario by 2030. However, this is a complex challenge given the country's heavy dependence on oil and gas hydrocarbons' production, transformation, and consumption, which contribute significantly to Qatar's gross domestic product and overall wealth. Qatar's economy is also heavily dependent on desalinated water, much like other Gulf Cooperation Council (GCC) countries in the region. This is because Qatar, like its neighboring countries, is located in an arid region with limited natural sources of freshwater. As a result, desalinated water is critical for meeting the country's domestic and industrial water needs.^[^ ^51^ ^]^ Aside from the insignificant amount of groundwater used for irrigation, the country mainly relies on the desalination of seawater to meet urban demands using energy‐intensive technologies (99% of municipal water is desalinated water). Currently, there are eight thermal seawater desalination plants, seven of which utilize thermal multi‐stage‐flash and one that employs the multi‐effect distillation technology.^[^ ^52^ ^]^ Both desalinated water and electricity are generated at independent power and water and producers solely driven by natural gas and then transmitted and distributed via the sole transmission and distribution system owner and operator in Qatar. As illustrated in **Table** **4**, the oil and gas industry is a major contributor to Qatar's gross domestic product, and hence its wealth. 90% of Qatar's exports were from oil and natural gas in May 2022.^[^ ^53^ ^]^ Hence, reducing emissions while sustaining economic activity in Qatar's economy is a multifaceted challenge that necessitates a cohesive and collaborative approach from various sectors and stakeholders.

**Table 4:** Qatar's energy and emissions recent figures^[^ ^54^, ^55^, ^56^ ^]^

GDP (2021) ‐ Billion US dollar	179.68	
Primary energy consumption (2021) [EJ]	1.93	Share of fossil fuels 99.98% : oil 25.38% and natural gas 74.6% Share of low carbon: less than 0.01%
CO_2_ emissions from energy (2021) ‐ Mt CO_2_ eq	115.3	
Electricity generation (2021) [TWh]	51.6	Share of fossil fuels 99.98% (natural gas)
Electricity consumption per capita (2021) [kWh]	17 005	Based on electricity net distribution

**Figure 3:**
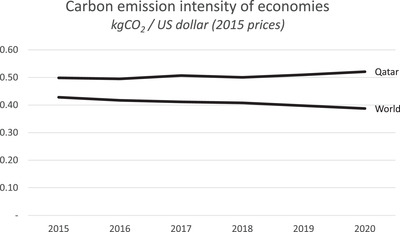
Carbon intensity of Qatar's economy.^[^ ^57^ ^]^

Qatar's carbon intensity was 0.52 kg CO_2_ per dollar of GDP in 2020,^[^ ^57^ ^]^ which is considerably higher than the global average as shown in **Figure** **3**. The country has made some efforts to reduce its carbon intensity through investment in renewable energy and energy efficiency, but these initiatives face significant challenges some of which are the absence of renewable energy sources in the energy mix as well as expansion of natural gas facilities and production capacity by 64% to meet the growing demand.^[^ ^58^ ^]^ Pertinently, it is important to consider the concept of the carbon intensity of the economy to assess the amount of emission required to produce a unit of economic output and the potential of emission reduction given the ongoing expansion of natural gas production facilities onshore and offshore. and therefore must be balanced against the need to reduce the carbon intensity of the economy.

### 4.1 Energy Production and Supply

**Figure 4:**
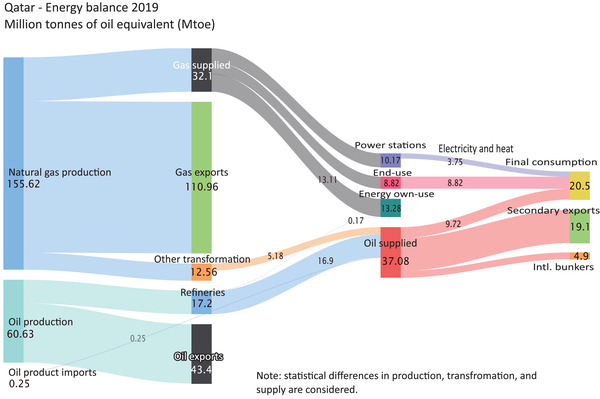
Qatar's energy balance—Sankey diagram 2019.^[^ ^59^, ^60^ ^]^

It is crucial to understand the broader context of Qatar's energy system and associated GHG emissions when designing and developing strategies for reducing emissions. The country's heavy reliance on fossil fuels is evident from its energy balance and final energy consumption, with fossil fuels accounting for over 99% of its energy production mix. **Figure** **4** shows that more than 70% of Qatar's natural gas output is exported as primary exports, while less than 10% undergoes further transformations and is then exported as secondary exports. The remaining 20% is supplied to the domestic market for energy conversion and final consumption. Natural gas is primarily used for power generation in Qatar, with a third of domestic natural gas supplies used for this purpose, and the rest is allocated to end‐use sectors such as buildings, transport, and industry. Furthermore, natural gas is used directly for energy and non‐energy purposes, such as a feedstock for different industries, with over 40% of the domestic natural gas supply used for the energy sector's own use in oil refining, oil and gas extraction, and other energy‐producing processes. Over 70% of the oil produced in Qatar is exported, with the rest split between final consumption, secondary exports, and a smaller portion for overseas bunkers.

Qatar's heavy reliance on fossil fuels for energy production, particularly in light of the fact that a significant portion of its energy production is exported, presents a critical challenge to reducing GHG emissions. Given that fossil fuels are the largest contributor to GHG emissions, reducing emissions from energy production is crucial not only for Qatar's own domestic environment but also for global climate mitigation efforts. Consequently, given this level of dependence and the existing oil and gas production infrastructure, Qatar will need to strategically plan for the potential for fossil fuel as the assets to become uneconomical or stranded and ultimately cope with “locking in carbon,” in the context of the low‐carbon transition, that is when fossil fuel‐intensive systems hinder or delay the transition to low‐carbon energy production alternatives.^[^ ^61^ ^]^ As such, it is imperative that Qatar prioritize the development and implementation of a comprehensive energy policy framework that accounts for the overarching dimensions of sustainable energy production and use.

### 4.2 Energy Consumption

**Figure 5:**
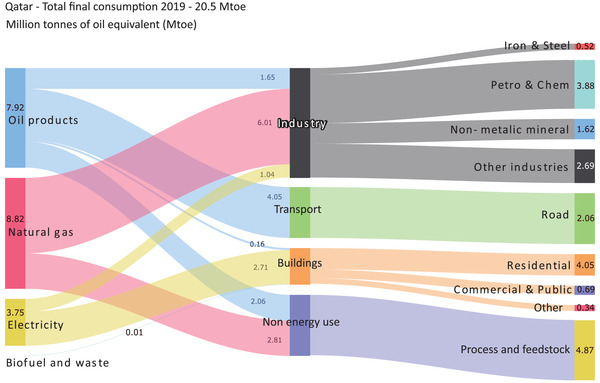
Qatar's final energy consumption 2019.^[^ ^59^, ^60^ ^]^

Qatar's final energy consumption is almost entirely met by domestic oil and gas production and electricity, as shown in **Figure** **5**. The industrial sector is the largest energy‐consuming sector, accounting for around 43% of the total delivered energy (20.5 Mtoe), followed by non‐energy use of fuels (natural gas and oil) and energy products as raw materials and feedstocks in different industries, which accounts for 23% of the total delivered energy. The transport sector accounts for 20% of the final energy consumed, with almost all of it coming from oil products. The building sector's energy demand accounts for 14% of the total final energy consumption, which is mainly met by electricity generated from natural gas power plants. The high energy consumption in Qatar's industrial sector, combined with the significant contribution of non‐energy use of fuels and feedstocks to the country's energy consumption, highlights the need for a comprehensive approach to energy efficiency and conservation in the industrial sector. Additionally, given that the transport sector is heavily dependent on oil products, the adoption of low‐carbon transport solutions such as electric vehicles and alternative fuels could significantly reduce Qatar's greenhouse gas emissions. The reliance on natural gas for electricity generation to meet the energy demands of the building sector also underscores the importance of diversifying Qatar's energy mix by increasing the share of renewable energy sources such as solar and wind power. Furthermore, investing in energy‐efficient buildings and encouraging the adoption of energy‐efficient practices in the building sector could help reduce the overall energy demand and promote a more sustainable built environment. Qatar has one of the highest per capita electricity consumption rates in the world due to a combination of factors such as the hot and humid climate and the significant energy use for space cooling, high standard of living, rapid industrial growth, population growth, subsidized electricity prices, and a lack of awareness about energy efficiency.^[^ ^56^ ^]^ These factors have led to significant energy consumption in the industrial, transport, and building sectors.

### 4.3 Energy‐Related GHG Emissions

**Figure 6:**
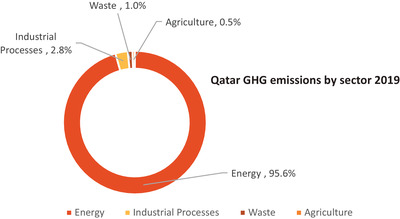
Qatar's GHG emissions by sector in 2019.^[^ ^35^, ^63^ ^]^

As illustrated in **Figure** **6**, energy and industrial processes account for 98.4% of Qatar's GHG emissions, whereas waste (wastewater and landfills) and agriculture account for an insignificant share.^[^ ^62^ ^]^ Natural gas and oil combustion are responsible for 80% of energy‐related GHG emissions across all sectors, with fugitive emissions accounting for the remaining 20% of emissions. Nearly a third of Qatar's total energy emissions are attributable to the country's energy sector's own use of fuel combustion. This demonstrates the necessity of low‐carbon natural gas and oil production in any planned decarbonization efforts.

**Figure 7:**
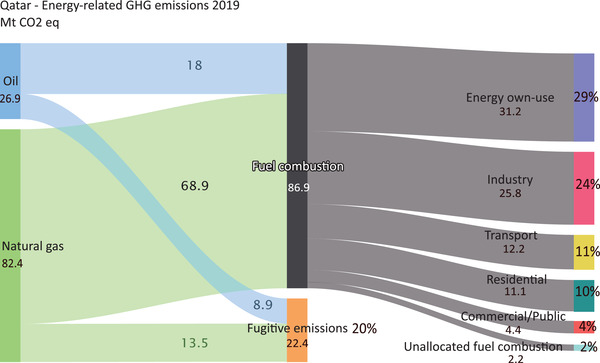
Qatar's GHG energy‐related emissions in 2019.^[^ ^35^, ^63^ ^]^

The country's GHG emissions profile gives Qatar a unique complexity not shared by many countries; emissions from the production of natural gas and the relatively modest oil production are dependent on both the emissions intensity of production itself and the global demand for natural gas that is to be met from exports. When GHG emissions from power generation are allocated to their respective end‐use sectors, the industrial sector accounts for more than a quarter of total energy‐related emissions, only being surpassed by the energy sector's own use as shown in **Figure** **7**. Transport and buildings (combined residential and commercial) were responsible for 12.2% and 15.5% of total energy emissions, respectively. As for electricity and heat generation collectively, prior to allocation to end‐use sectors, accounted for 23.9 Mtoe (22% of Qatar's energy‐related GHG emissions). One intriguing aspect of Qatar's emissions profile is that methane accounts for 18% of energy‐related emissions, which is significantly higher than methane's global 10% share of GHG emissions. This is because, like some other GCC nations, its domestic energy consumption is relatively low and the vast majority of its production is destined for export.

## 5 Policy Implications

Our analysis of the selected national LEDS structure and their corresponding mitigation measures, as well as examining the distinctive characteristics of Qatar's energy production and consumption and the energy‐related emissions, has essential elements that require attention when formulating Qatar's LEDS. Nevertheless, the prospect of energy transition in major oil and gas producing/exporting nations, as in the case of Qatar, has been commonly viewed as posing potential negative impacts on their economies due to their reliance on fossil fuels. In this regard, Qatar is urged to establish a decarbonization pathway for its energy sector that is pragmatic, while also incorporating measures aimed at mitigating GHG emissions without compromising its energy security and economic growth. A well‐designed LEDS could help Qatar to identify potential pathways for transitioning to a low‐carbon economy while balancing economic growth and environmental sustainability. It could also enable decision‐makers in Qatar to evaluate the various enablers and obstacles, risks, and opportunities associated with different emission reduction options and guide investments in sustainable technologies and infrastructure. The present study's findings have far‐reaching implications for the design, process, and implementation of LEDS in Qatar as discussed in what follows.

### 5.1 Alignment with the NDCs Is an Ongoing Policy Exercise

Meeting Qatar's NDCs is the first step in any viable long‐term strategy for reaching a more ambitious emissions reduction objective. Time horizons for Qatar's NDC are around 2030, and most of the previously mentioned national policies and strategies, are the same or shorter. In essence, this relationship is reciprocal since NDCs provide the assurance gauge for assessing the implementation of long‐term energy‐related emission reduction through its short‐term targets.^[^ ^64^ ^]^ Conversely, Qatar using its forthcoming LEDS can establish a framework for investigating the implications of short‐term actions and mitigation measures on the long‐term transformation necessary for deep decarbonization of energy sectors. Several countries had either explicitly indicated that their long‐term strategies are already aligned with their submitted NDC or highlighted the need to update their NDCs accordingly.

### 5.2 Modeling of Long‐Term Scenarios of Energy Systems

Developing solid and all‐encompassing long‐term emission strategies is facilitated by the use of rigorous analytical frameworks and tools, usually models of mitigation scenarios. Several of the 51 strategies already submitted to UNFCCC included the outputs of mitigation modeling exercises, providing alternative pathways for countries to reach their long‐term emissions targets and highlighting opportunities and trade‐offs associated with these models. These pathways map out the entire period of time between the present and the time for which a goal is set, with the intention of determining what actions make the most sense right now in the context of ultimately achieving that emission reduction goal. Ideally, “backcasting” rather than forecasting should be used to link the desired end‐state to the present when constructing a set of potential pathways. Given the inherent uncertainty of long‐term modeling, these models are not intended to be exact forecasts or prescriptions of future events; rather, they are intended to show what is feasible. The findings of these exercises are critical for informing decision‐makers about the policies and investments that must be made today to guarantee Qatar is on track to reach emission reduction targets. However, because models are often simplifications of real‐world energy systems, it is critical to account for their inherent uncertainty when making key decisions based on them. Regulatory, institutional, or other structural changes can be identified after pathways are laid out in detail, allowing for the formulation of specific recommendations feeding into the long‐term strategy.

### 5.3 Sector‐Specific Emission Reduction Targets

The national climate change action plan for 2030 includes sector‐specific emission reduction targets of 37 Mt CO_2_ equivalent. However, long‐term emission reduction goals entail an in‐depth analysis of potential prospects across different sectors. It is necessary to fully comprehend the magnitude of investment requirements, technology uptake, emissions reduction, and market shift trajectories. This helps identify both pathways for reducing energy intensity and GHG emissions. Marginal abatement cost curves can be developed to synthesize sector‐specific targets and yield marginal abatement costs for a variety of mitigation strategies.^[^ ^65^, ^66^ ^]^ Additionally, these sector‐specific targets can also help to mobilize public and private sector support for emissions reduction efforts. By setting realistic and achievable targets for specific sectors, policymakers can engage with businesses, industry associations, and civil society organizations to develop strategies to achieve these targets.

### 5.4 Emission Intensity Indicators

Literature has shown that comprehensive analysis of these indicators can provide insights into a country's emissions profile and energy system efficiency. Given its distinct economic dynamics and emission indicators; carbon intensity, energy intensity, emissions per capita, and so on, Qatar would necessarily study the relationship between these indicators that are useful for tracking progress and benchmarking. Research and policy‐makers may use analytical approaches including statistical and econometric modeling and analysis and evaluate the potential impact of different policy interventions. Not only is it essential for Qatar to take emissions intensity indicators into account when making a long‐term low‐emission development strategy, but it is also equally important for fostering economic growth that is not tied to emissions; economic growth decoupling

### 5.5 Options for Decarbonizing Energy Production and Consumption

The long‐term strategies studied in this research echoed countries' interest in evaluating different routes and leveraging the potential role of advanced clean technologies consistently identified in global energy transition scenarios and the reduction of energy‐related GHG emissions. However, the degree to which each technology and emissions reduction alternative is adopted varies according to factors such as cost, technological maturity, policy preference, market, and national circumstances. By the same token, to develop a successful long‐term low‐emission strategy, Qatar must consider a range of decarbonization options that are suitable for its specific context. These may include a combination of renewable energy technologies, energy efficiency measures, and carbon capture and storage technologies for fossil fuel‐based industries. The adoption of decarbonization options in Qatar must also take into account the country's existing infrastructure, institutional capacity, and regulatory frameworks. The government of Qatar has taken steps to promote renewable energy, including the installation of solar panels on public buildings and the establishment of several solar power plants. However, there is significant potential for further expansion of renewable energy in Qatar given its abundant solar irradiation in particular. Fugitive emissions are an important aspect of any long‐term low‐emission strategy for Qatar, given their high share in the country's total emissions. Fugitive emissions refer to the unintentional release of GHG into the atmosphere during the production, processing, transportation, and storage of oil and gas. The high share of fugitive emissions in Qatar's emission profile is a significant challenge that needs to be addressed in a comprehensive approach. This approach should include strengthening regulations, improving monitoring and reporting, investing in new technologies, and promoting best practices in the oil and gas sector to achieve meaningful reductions in fugitive emissions in Qatar.

### 5.6 Governance and Stakeholder Engagement

It is clear that governance difficulties will play a critical role in deciding whether or not long‐term emission development programs succeed. Weak leadership can lead to strategies being ignored, mistrusted, or created without sufficient input from the public, business sector, and civil society at large. Lack of collaboration amongst relevant parties is to blame for the ineffectiveness and gaps in current attempts to reduce emissions related to the energy sector. To ensure the long‐term success of Qatar's effort to decrease emissions, the country should codify its goals into law, providing the required strong backing and triggering for a concerted governmental response. The development and subsequent roll‐out of Qatar's long‐term emission development strategy should be mapped to Qatar's current institutional structure to specify the coordination and interface areas and identify any necessary changes. It is crucial that stakeholders be actively involved in the development LEDS. Expert opinions and knowledge gathered through stakeholder engagement complement and augment quantitative, analytical models described earlier when developing long‐term strategies.

### 5.7 Building on What Already Exists

The necessity to integrate Qatar's existing short‐term national vision, strategies and plans to the development of a long‐term emission development strategy expands on prior efforts and offers a substantial framework for achieving more aggressive emission reduction goals. Four essential national visionary, strategy and policy documents serve as the cornerstone for Qatar's LEDS development: *Qatar's National Vision* 2030^[^ ^67^ ^]^ plans for development along four interrelated axes: human, social, economic, and environmental development; *The National Development Strategy* (2018–2022)^[^ ^68^ ^]^ presented in continuation of previous strategies aims at achieving the aspiration of national vision and advocating, among other things, diversification of the energy mix and investment in renewable energy; *Qatar Energy's updated sustainability strategy* unveiled in 2021 reaffirms the commitment to cleaner production across oil and gas value chain;^[^ ^69^ ^]^ and finally *National Climate Change Action Plan* 2030^[^ ^70^ ^]^ recently approved by the cabinet coinciding with the debut of COP26 in Glasgow. The plan communicates Qatar's most recent stance on climate change, which includes 36 mitigation strategies spanning the energy supply and end‐use sectors. While these national documents are ostensibly established to carry out short‐term to medium goals, in practice they enable longer‐term, more profound long‐term shifts.

## 6 Conclusions, Limitations, and Future Directions

Given the complexity of what fulfilling the Paris Agreement challenge would entail, Qatar will need a long‐term low‐emission development strategy. One of the main advantages of the LEDS, in the eyes of many stakeholders, is its capacity to focus short‐term efforts. The notion of using LEDS as the centerpiece of a more comprehensive set of climate policies is gaining traction. They identify the long‐term, systemic change required to mitigate and adapt to climate change. Qatar's stance on climate change, as well as any proposed transformation strategy, are strongly related to its energy system. Therefore, Qatar must create, plan, and seek future energy scenarios that require few to no trade‐offs with other national development ambitions and policy goals as it moves toward decarbonization.

This study is an essential addition to the existing literature on emissions reduction and energy transition in Qatar, as it underscores the necessity for further research and discussion toward identifying sustainable and feasible transformation strategies for Qatar's energy system. This paper acknowledges several limitations in the context of developing long‐term low‐emission development strategies. Although the study provides valuable insights, it focuses only on the energy sector and excludes other important sectors that contribute to GHG emissions in Qatar. While the waste and agriculture sectors have a trivial contribution to the total emissions in Qatar, the emissions from industrial processes are significant and warrant consideration in any future related work. Additionally, the analysis is limited to mitigation measures in the context of climate action and excludes adaptation measures from the scope. A more comprehensive approach should also include adaptation measures addressing the unavoidable impacts of climate change, in addition to mitigation measures across all sectors.

Based on the findings of our study, we suggest several potential avenues for future research directions. One compelling area of study is the use of integrated assessment modeling (IAM) framework and CGE models to investigate potential deep decarbonization and transformation routes in Qatar's energy system in a holistic fashion. IAM integrates energy, climate, and economics modules to optimize policy evaluation. Using historical data and theoretical frameworks, CGE models can “play out” what might happen to an economy if certain policies, technologies, or other factors were altered. These models, whether generic in scope or tailored to Qatar's needs, assist in setting priorities for mitigation efforts and revealing the opportunities and trade‐offs typically associated with them. Furthermore, planning for a carbon‐neutral or low‐carbon energy supply through the deployment of alternative energy carriers is another important area of research for Qatar. Existing literature has shown that electricity and hydrogen would be the primary sources of energy in carbon‐neutral energy systems. Low‐carbon transition is futile in the absence of adequate infrastructure planning given the extent of the capacity requirements and the rate at which it would need to be installed. The level of infrastructure change necessary in Qatar's energy sector is a significant challenge. Hence, it requires energy planning with an adequate spatial and temporal resolution that captures and projects infrastructure requirements with a particular emphasis on the coupling of renewable and hydrogen in power systems. Diversification of energy resources is important for Qatar. It is timely and warranted to conduct comprehensive research into the potential of leveraging circular economy concepts, determining the economic viability of second‐generation biofuels, particularly waste‐based biofuels that can be derived from either municipal or industrial waste, and determining the impact of policies on the expansion of Qatar's bioenergy sector. Finally, tar's emissions trajectories toward a low‐carbon energy system are mostly dictated by oil and gas strategies for primary energy production, the majority of which is exported. More research is required to determine whether it is feasible to integrate low‐carbon technologies into the oil and gas production process, deploy renewable energy sources, assess the costs and benefits of retrofitting CCUS technologies, replace some of the natural gas used with hydrogen, and most importantly, identify ways to reduce methane emissions throughout the entire value chain.

## Conflict of Interest

The authors declare no conflict of interest.
